# Potentially Functional Polymorphism in IL-23 Receptor and Risk of Acute Myeloid Leukemia in a Chinese Population

**DOI:** 10.1371/journal.pone.0055473

**Published:** 2013-02-05

**Authors:** Xifeng Qian, Songyu Cao, Guohua Yang, Yun Pan, Chenyu Yin, Xiang Chen, Ying Zhu, Yun Zhuang, Yunfeng Shen, Zhibin Hu

**Affiliations:** 1 Department of Hematology, Wuxi People’s Hospital Affiliated to Nanjing Medical University, Wuxi, China; 2 Department of Epidemiology and Biostatistics and Ministry of Education Key Lab for Modern Toxicology, School of Public Health, Nanjing Medical University, Nanjing, China; 3 Wuxi People’s Hospital Affiliated to Nanjing Medical University, Wuxi, China; 4 Jiangsu Key Lab of Cancer Biomarkers, Prevention and treatment, Cancer Center, Nanjing Medical University, Nanjing, China; Institut national de la santé et de la recherche médicale (INSERM), France

## Abstract

The interleukin-23 (IL-23) and its receptor (IL-23R) mediate the direct antitumor activities in human hematologic malignancies including pediatric acute leukemia. Two potentially functional genetic variants (*IL-23R* rs1884444 T>G and rs6682925 T>C) have been found to contribute to solid cancer susceptibility. In this study, we conducted a case-control study including 545 acute myeloid leukemia (AML) patients and 1,146 cancer-free controls in a Chinese population to assess the association between these two SNPs and the risk of AML. We found that *IL-23R* rs1884444 TG/GG and rs6682925 TC/CC variant genotypes were associated with significantly increased risk of AML [rs1884444: adjusted odds ratio (OR) = 1.28, 95% confidence interval (CI) = 1.01–1.62; rs6682925: adjusted OR = 1.30, 95%CI = 1.01–1.67], compared to their corresponding wild-type homozygotes, respectively. These findings indicated that genetic variants in *IL-23R* may contribute to AML risk in our Chinese population.

## Introduction

Acute myeloid leukemia (AML), a cancer of the myeloid line of blood cells, is the most common acute leukemia affecting adult [1]. The abnormal white blood cells accumulate in the bone marrow, thus interfering with the production of normal blood cells. Although most cases of AML have no obvious cause, it is considered that several factors may play a role in the increased occurrence of AML, including chemical exposure [2], [3], ionizing radiation [4], and genetics [5].

Interleukin (IL)-23 is a heterodimeric proinflammatory cytokine composed of a p19 subunit and a p40 subunit, which is also part of IL-12 [6], [7]. IL-23 is predominantly produced by antigen-presenting cells in response to microbial or host immune stimuli and is involved in the regulation of immune responses against infections and tumor development through the engagement of the IL-23 receptor (IL-23R) [8]. Recent studies also showed the direct antitumor activities of IL-23/IL-23R in human hematologic malignancies, i.e., pediatric acute leukemia, and IL-23 directly dampens tumor growth in vitro and in vivo through the inhibition of tumor cell proliferation and induction of apoptosis [9], [10]. The *IL-23R* gene located on chromosome 1p31 encodes one subunit of the IL-23R [11].Two potentially functional common variants of *IL-23R*, rs6682925 (T>C) located at 907-bp upstream from the transcriptional start position, and rs1884444 (T>G) located at codon 3 with amino acid His substituted by Gln in exon 2, have been shown to have association with risk of several solid cancers [12]–[14]. Here, we hypothesized these two single-nucleotide polymorphisms (SNPs) may also be associated with risk of AML.

## Results

Of the 545 AML cases, 291 (53.4%) were males and the mean age at diagnosis was 44.1±17.2 years old, while among the 1146 cancer-free controls, 820 (71.5%) were males and the mean age was 59.0±9.7 years old. The clinical and cytogenetic characteristics of the AML cases are summarized in Table 1.

**Table 1:** Clinical Characteristics of AML Patients.

Characteristics	AML (n = 545)	
	No.	(%)
**Lineage**		
Myeloid	378	69.4
Myeloid and Lymphoid	157	28.8
Myeloid and Monocytic	10	1.8
**Classification of diagnosis**		
M0	3	0.6
M1	103	18.9
M2	175	32.1
M3	90	16.5
M4	58	10.6
M5	89	16.3
Unknown	27	5.0
**Karyotype**		
Aberrant	246	45.1
t(9;22)	11	2.0
t(8;21)	48	8.8
t(15;17)	64	11.7
complex	114	20.9
others	9	1.7
Normal	235	43.1
Unknown	64	11.8
**Molecular subtype**		
Common fusion gene transcripts	187	34.3
PML/RARα	77	14.1
BCR/ABL	13	2.4
AML1/ETO	54	9.9
CBFβ/MYH11	10	1.8
MLL rearrangement	15	2.8
WT1	11	2.0
Others	7	1.3
No common fusion gene ranscripts	303	55.6
Unknown	55	10.1

Genotyping was successfully performed in 537 cases and 1137 controls for rs1884444 (call rate = 99.0%) and 525 cases and 1117 controls for rs6682925 (call rate = 97.1%). The genotype distributions of these two variants between the cases and controls are shown in Table 2. The observed genotype frequencies for both variants were in Hardy-Weinberg equilibrium among the controls (*P* = 0.829 for rs1884444 and *P* = 0.686 for rs6682925). Multivariate logistic regression analysis showed that the rs1884444 TG and combined genotypes TG/GG were associated with a significantly increased risk of AML (adjusted OR = 1.34, 95%CI = 1.04–1.72 for TG; adjusted OR = 1.28, 95% CI = 1.01–1.62 for TG/GG), compared with the rs1884444 wild-type TT, respectively. Similarly, compared to the wild TT genotype, rs6682925 CC and combined TC/CC genotypes contributed to AML risk by 1.48-fold (95% CI = 1.03–2.13) and 1.30-fold (95% CI = 1.01–1.67), respectively.

**Table 2:** Distribution of Alleles and Genotypes of *IL-23R* Variants and Their Association with AML Risk.

	AML (n = 545)		Controls (n = 1146)		OR (95%CI)^a^	*P* a	OR (95%CI)^b^	*P* b
	N	%	N	%				
**rs1884444 T>G** c								
TT	223	41.5	519	45.7	1.00		1.00	
TG	254	47.3	496	43.6	1.19(0.96, 1.48)	0.115	1.34(1.04, 1.72)	0.024
GG	60	11.2	122	10.7	1.15(0.81, 1.62)	0.445	1.05(0.70, 1.57)	0.814
TG/GG	314	58.5	618	54.3	1.18(0.96, 1.46)	0.113	1.28(1.01, 1.62)	0.047
G allele	34.8%		32.5%		1.11(0.95, 1.29)	0.189	1.12(0.94, 1.34)	0.214
**rs6682925 T>C** d								
TT	184	35.0	452	40.5	1.00		1.00	
TC	247	47.1	522	46.7	1.16(0.93, 1.46)	0.197	1.25(0.96, 1.62)	0.103
CC	94	17.9	143	12.8	1.62(1.18, 2.21)	0.003	1.48(1.03, 2.13)	0.033
TC/CC	341	65.0	665	59.5	1.26(1.02, 1.56)	0.036	1.30(1.01, 1.67)	0.039
C allele	41.4%		36.2%		1.25(1.07, 1.45)	0.004	1.22(1.03, 1.45)	0.022

a Crude odds ratio (OR), 95% confidence interval (CI) and corresponding *P* value.

b Adjusted for age and gender.

c Genotypes were unavailable in 8 cases and 9 controls.

d Genotypes were unavailable in 20 cases and 29 controls.

Stratified analyses of these two SNPs are summarized in Table 3. No significant heterogeneity between the subgroups was detected for rs6682925. For rs1884444, the increased risk of combined TG/GG genotypes was more significant in older patients (more than 45 years old at diagnosis), and *P* value for the heterogeneity was 0.030. However, there was no interaction between rs1884444 and age on AML risk (*P* = 0.097).

**Table 3:** Stratification Analysis of rs1884444 and rs6682925 Genotypes by Selected Variables in AML Patients and Controls.

Characteristics	rs1884444						rs6682925					
	Case		Control		OR(95%CI)	*P* d	Case		Control		OR(95%CI)	*P* d
	TT	TG/GG	TT	TG/GG			TT	TC/CC	TT	TC/CC		
**gender**												
Male	115	172	365	448	1.30(0.95, 1.78)^a^	0.848	98	183	318	483	1.26(0.91, 1.74)^a^	0.745
Female	108	142	154	170	1.24(0.86, 1.80)^a^		86	158	134	182	1.37(0.93, 2.01)^a^	
**Age at diagnosis, year**												
≤45	134	159	48	67	0.85(0.55, 1.32)^b^	0.030	109	179	41	74	0.91(0.58, 1.43)^b^	0.062
>45	89	155	471	551	1.52(1.14, 2.04) b		75	162	411	591	1.53(1.13, 2.08)^b^	
**Lineage**												
Myeloid	158	215	519	618	1.21(0.93, 1.59)^c^	0.985	131	234	452	665	1.25(0.94, 1.65)^c^	0.890
Myeloid and Lymphoid	61	93	519	618	1.26(0.86, 1.83) c		49	101	452	665	1.32(0.89, 1.95)^c^	
Myeloid and Monocytic	4	6	519	618	1.20(0.34, 4.30) c		4	6	452	665	0.96(0.27, 3.44)^c^	
**Karyotype**												
t(8;21)	22	26	519	618	0.94(0.51, 1.73) c	0.726	15	31	452	665	1.29(0.67, 2.48)^c^	0.740
t(15;17)	23	39	519	618	1.34(0.74, 2.42) c		21	42	452	665	1.26(0.69, 2.29)^c^	
Complex	53	60	519	618	0.93(0.61, 1.40) c		44	63	452	665	0.91(0.59, 1.39)^c^	
Others	8	11	519	618	1.05(0.41, 2.69) c		6	13	452	665	1.34(0.50, 3.63)^c^	
Normal	95	136	519	618	1.27(0.92, 1.74) c		78	148	452	665	1.31(0.94, 1.82)^c^	
Unknown	22	42					20	44			–	
**Molecular subtype**												
PML/RARα	26	50	519	618	1.55(0.90, 2.69) c	0.647	23	51	452	665	1.37(0.78, 2.43)^c^	0.922
AML1/ETO	25	29	519	618	0.94(0.53, 1.66) c		19	33	452	665	1.10(0.60, 2.01)^c^	
Others	24	32	519	618	1.08(0.61, 1.91) c		21	34	452	665	1.06(0.59, 1.90)^c^	
No common fusion gene ranscripts	126	170	519	618	1.17(0.88, 1.56) c		102	188	452	665	1.23(0.92, 1.66)^c^	
Unknown	22	33					19	35				

a Adjusted for age.

b Adjusted for gender.

c Adjusted for age and gender.

d *P* value for heterogeneity test based on χ^2^-based Q test.

As the fusion gene AML1/ETO and PML/RARαrespectively accounted for the majority of molecular subtypes of M2 AML and M3 AML, we then investigated the association of these two genes and SNPs of *IL-23R* in M2 AML and M3 AML. However, there was no significant associations between AML1/ETO or PML/RARα and *IL-23R* variants in M2 and M3 AML, respectively, as shown in Table 4.

**Table 4:** Association of AML1/ETO and *IL-23R* Variants in M2 AML, and Association of PML/RARα and *IL-23R* Variants in M3 AML.

Classification	Molecularsubtype	rs1884444						rs6682925					
		Case		Control		OR(95%CI)^a^	*P* b	Case		Control		OR(95%CI)^a^	*P* b
		TT	TG/GG	TT	TG/GG			TT	TC/CC	TT	TC/CC		
M2	AML1/ETO	20	25	519	618	1.04(0.59, 1.83)	0.709	14	29	452	665	1.21(0.67, 2.20)	0.929
	No commonfusion generanscripts	51	55	519	618	0.91(0.60, 1.37)		35	66	452	665	1.17(0.76, 1.81)	
	Unknown & Others	8	14	519	618	–		7	14	452	665	–	
M3	PML/RARα	26	49	519	618	1.51(0.87, 2.62)	0.241	23	50	452	665	1.33(0.75, 2.37)	0.511
	No commonfusion generanscripts	5	4	519	618	0.64(0.17, 2.41)		4	5	452	665	0.82(0.22, 3.10)	
	Unknown & Others	3	1	519	618	–		3	1	452	665	–	

a Adjusted for age and gender;

b *P* value for heterogeneity test based on χ^2^-based Q test.

## Discussion

IL-23 is a proinflammatory heterodimeric cytokine sharing common subunits with IL-12 [6], [7], which could directly inhibit human acute myeloid leukemia cell growth [15]. With the similar structures and biological activities of IL-12, IL-23 may also have some effects on human malignancies, whereas its antitumor activity remains controversial. Some groups held the opinion that IL-23 can impair CD8^+^ T-lymphocyte-mediated immune surveillance and promote *de novo* carcinogenesis and tumor neovascularization [16]–[18]. In contrast, other groups have shown that IL-23 exerts antitumor activity through stimulation of T cells and NK cells [19]–[24]. IL-23 mediates these cancer-related effects through IL-23R. Cocco et al. demonstrated that IL-23/IL-23R acts as antitumor agent on hematologic malignancies including childhood acute leukemia [9], [10]. Recently, several studies evaluated the association between two variants (rs1884444 and rs6682925) of *IL-23R* polymorphisms and risk of solid cancers in Chinese population [12]–[14]. Xu et al. [12] reported that the variant alleles of rs6682925 and rs1884444 both increased the hepatocellular carcinoma risk. Chu et al. [13] found that rs6682925 TC/CC and rs1884444 TG/GG variant genotypes were associated with significantly increased risk of esophageal cancer. But Chen et al. [14] found the protective effect of rs1884444 G allele in gastric cancer. In this study, we found that the variant alleles of rs1884444 (T>G) and rs6682925 (T>C) were associated with an increased risk of AML in a Chinese population, although this type-specific association needs to be confirmed in other studies. However, up to date, only 3 studies focused on the association of rs1884444 with human diseases in non-Chinese population [25]–[27], and none of them were involved in leukemia. Additionally, there haven’t been any studies focused on rs6682925 in other population except Chinese. So we could not evaluate the presence of these SNPs in non-Chinese population.

Thus far, it is challenging to know the exact mechanisms of these two variants on AML carcinogenesis. The SNP rs1884444 locates at exon 2 of *IL-23R*, which is responsible for the signal peptide of IL-23R [28]. According to the wed-based prediction tool of PupaSuite2 (http://pupasuite.bioinfo.cipf.es/) [29], the T-to-G base change of rs1884444 may disrupt an exonic splicing enhancer, resulting in exon skipping, malformation, or transcript alternative splicing. Another possible explanation is that rs1884444 resulted in amino acid change (His>Gln), which may influence the ligand-receptor binding specificity and affinity, and thus modulate the proinflammatory effect by Th17 cell and get involved in the development of AML [14]. The SNP rs6682925 locates at 907-bp upstream from the transcriptional start position of *IL-23R.* The web-based tool of TFSEARCH 1.3 (http://www.cbrc.jp/research/db/TFSEARCH.html) [12] showed that the T-to-G base change of rs6682925 might affect a predicted GATA-X transcription factor binding, which may be involved in tumor differentiation and other carcinogenesis-related pathways [30], [31]. Xu et al. found that the C allele (G allele in antisense strand) of rs6682925 may increase the promoter activity of *IL-23R* by luciferase assay, which was consistent with the web-based prediction [12].

There are a few biases that may lead to spurious findings. Firstly, cases and controls were not matched on age and gender, but we used further statistical adjustment to minimize potential biases. Secondly, some risk predictors of AML, such as exposure to carcinogens and development of myelodysplasia, were not included in this analysis. Therefore, large population-based prospective studies with ethnically diverse populations are warranted to further elucidate the impact of *IL-23R* SNPs on AML susceptibility.

## Materials and Methods

### Ethics Statement

The study is in compliance with the Helsinki declaration, and was approved by the institutional review board of Nanjing Medical University. The informed written consent was obtained from all subjects.

### Study Subjects

All of the cases and controls were unrelated ethnic Han Chinese. The 545 newly diagnosed AML patients were consecutively recruited between December 2007 and August 2011 from Wuxi people’s Hospital Affiliated to Nanjing Medical University. There were no restrictions in terms of age, stage of disease, or histology preventing people from participating in the study. The 1,146 controls were randomly selected from those participating in the community screening of non-communicable disease conducted in Jiangsu Province during the same period as the cases were recruited and had no self-reported cancer history. At the time of recruitment, informed consent was obtained from each subject, and each subject provided 5 ml of venous blood.

### Laboratory Assays

Genomic DNA was extracted from a leukocyte pellet by traditional proteinase K digestion followed by phenol-chloroform extraction and ethanol precipitation. SNPs rs6682925 T>C and rs1884444 T>G were genotyped by using the TaqMan allelic discrimination assay on an ABI 7900 system (Applied Biosystems, Foster city, CA). The primers and probes for rs6682925 and rs1884444 were described previously [13].Genotyping was performed without knowing the subjects’ case or control status. Two blank (water) wells in each 384-well plate were used for quality control and more than 10% samples were randomly selected to repeat, yielding a 100% concordant.

### Statistical Analysis

The χ^2^ tests were used to evaluate differences in the distributions of genotypes of the two variants between the cases and controls. Hardy-Weinberg equilibrium was tested by a goodness-of-fit χ^2^ tests to compare the observed genotype frequencies to the expected ones among the control subjects. The associations between *IL-23R* SNPs and AML risks were estimated by computing the odds ratio (ORs) and their 95% confidence intervals (CIs) from both univariate and multivariate logistic regression analysis. Homogeneity test among different strata according to selected variables was assessed with the χ^2^-based Q tests. All the statistical analyses were performed with SAS 9.1.3 software (SAS Institute, Cary, NC). *P*<0.05 was the criterion of statistical significance, and all statistical tests were two sided.
